# Characterization of Microbiota in Bronchiectasis Patients with Different Disease Severities

**DOI:** 10.3390/jcm7110429

**Published:** 2018-11-09

**Authors:** Sang Hoon Lee, YeonJoo Lee, Jong Sun Park, Young-Jae Cho, Ho Il Yoon, Choon-Taek Lee, Jae Ho Lee

**Affiliations:** 1 Division of Pulmonology, Department of Internal Medicine, Severance Hospital, Institute of Chest Diseases, Yonsei University College of Medicine, Seoul 120-752, Korea; tearpoem9@gmail.com; 2 Division of Pulmonary and Critical Care Medicine, Department of Internal Medicine Seoul National University Bundang Hospital, 82 Gumi-ro, 173 Beon-gil, Bundang-gu, Seongnam-si, Gyeonggi-do 463-707, Korea; yjlee1117@snubh.org (Y.L.); jspark.im@gmail.com (J.S.P.); lungdrcho@gmail.com (Y.-J.C.); dextro70@gmail.com (H.I.Y.); ctlee@snu.ac.kr (C.-T.L.)

**Keywords:** bronchiectasis, FACED score, microbiome

## Abstract

The applications of the 16S rRNA gene pyrosequencing has expanded our knowledge of the respiratory tract microbiome originally obtained using conventional, culture-based methods. In this study, we employed DNA-based molecular techniques for examining the sputum microbiome in bronchiectasis patients, in relation to disease severity. Of the sixty-three study subjects, forty-two had mild and twenty-one had moderate or severe bronchiectasis, which was classified by calculating the FACED score, based on the FEV_1_ (forced expiratory volume in 1 s, %) (F, 0–2 points), age (A, 0–2 points), chronic colonization by *Pseudomonas aeruginosa* (C, 0–1 point), radiographic extension (E, 0–1 point), and dyspnoea (D, 0–1 point). Bronchiectasis was defined as mild, at 0–2 points, moderate at 3–4 points, and severe at 5–7 points. The mean age was 68.0 ± 9.3 years; thirty-three patients were women. *Haemophilus* (*p* = 0.005) and *Rothia* (*p* = 0.043) were significantly more abundant in the mild bronchiectasis group, whereas *Pseudomonas* (*p* = 0.031) was significantly more abundant in the moderate or severe group. However, in terms of the alpha and beta diversity, the sputum microbiota of the two groups did not significantly differ, i.e., the same dominant genera were found in all samples. Further large-scale studies are needed to investigate the sputum microbiome in bronchiectasis.

## 1. Introduction

Bronchiectasis is a chronic, irreversible airway disease with abnormal dilatation of one or more bronchi, causing chronic cough and purulent sputum production. Impaired mucociliary clearance in bronchiectasis patients is associated with continuous or repeated respiratory infection, inducing a vicious cycle of blockage, inflammation, exacerbation, and damage in the affected bronchi [1]. Bronchiectasis is associated with extended hospitalizations and high mortality, causing a significant economic burden [2,3].

Prevention of exacerbation, reduction of respiratory symptoms, and stopping the progression of the disease are important for the management of bronchiectasis [4]. By improving the bronchial hygiene and decreasing bronchial inflammation, recurrent infection and frequent exacerbation can be prevented [5]. Therefore, the ability to precisely identify colonizing bacterial species, including potential pathogens, is important for clinicians who treat bronchiectasis patients.

Conventional, culture-based microbiological analyses identified multiple bacterial pathogens in bronchiectasis patients, such as *Pseudomonas aeruginosa*, *Haemophilus influenzae*, *Streptococcus pneumoniae*, *Staphylococcus aureus*, and *Moraxella catarrhalis*. Importantly, previous studies showed that the *P. aeruginosa* colonization in bronchiectasis was linked to clinical, functional, and radiographic deterioration. Although standard culture-based diagnostic methods are widely used, chronic infections caused by anaerobes or certain bacterial species that barely grow under standard conditions are difficult to diagnose using these methods [6]. The application of next generation sequencing (NGS), using 16S rRNA gene pyrosequencing has expanded our understanding of the pathogenesis of bronchiectasis and is helping physicians to select appropriate antibiotic treatments [7].

Martínez-García et al. used five dichotomized variables to develop a scoring system for non-cystic fibrosis bronchiectasis, known as the “FACED score”, which considers lung function, age, colonization by *P. aeruginosa*, radiographic extension, and dyspnoea [8]. The authors conducted a multicenter, observational study, with eight hundred and nineteen bronchiectasis patients who were classified according to disease severity, in relation to the five-year all-cause mortality.

In this study, we employed culture-independent, DNA-based molecular techniques for examining the composition of the bacterial microbiota in sputum samples, in relation to disease severity, which we derived using the FACED scoring system.

## 2. Experimental Section

### 2.1. Study Population

**Figure 1:**
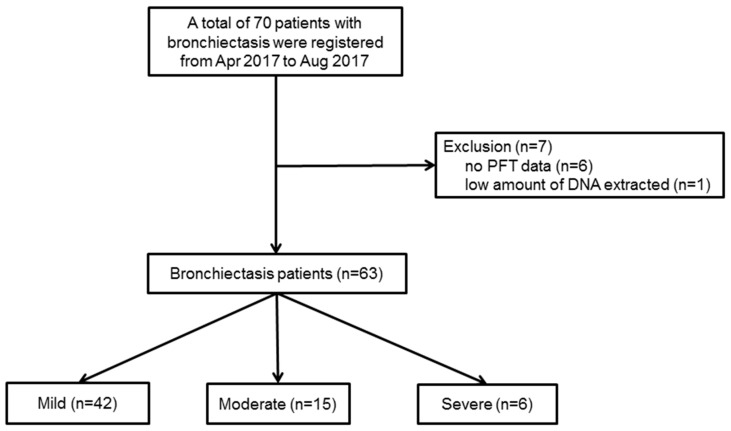
Patient flow chart. From 1 April 2017 to 31 August 2017, a total of seventy patients with bronchiectasis agreed to participate in this prospective study, but seven patients were excluded from this study because of incomplete data (*n* = 6) or an insufficient quantity of DNA extracted for the analysis (*n* = 1). PFT, pulmonary function test.

Bronchiectasis was diagnosed by high-resolution computed tomography (HRCT). Patients who had active tuberculosis or trauma/tuberculosis-related destroyed lungs, were excluded from the study. Figure 1 shows the patient flow chart. Initially, from 1 April 2017 to 31 August 2017, a total of seventy patients with bronchiectasis agreed to participate in this prospective study, but seven patients were excluded from the study because of incomplete data (*n* = 6), or a low quantity of DNA extracted for the analysis (*n* = 1). Therefore, a total of sixty-three patients with bronchiectasis were investigated in this study.

The severity of bronchiectasis was classified using the FACED score as follows; percentage of predicted forced expiratory volume in 1 s (FEV_1_ in %) (F, cut-off 50%, 0–2 points); age (A, cut-off 70 years, 0–2 points); presence of chronic colonization by *P. aeruginosa* (C, dichotomic, 0–1 point); radiographic extension (E, number of lobes affected, cut-off two lobes, 0–1 point); and dyspnoea (D, cut-off grade II on the Medical Research Council scale, 0–1 point). Mild bronchiectasis was defined as 0–2 points, moderate was 3–4 points, and severe was 5–7 points [8]. Out of the sixty-three patients, forty-two had mild bronchiectasis, and twenty-one had moderate (*n* = 15) or severe (*n* = 6) bronchiectasis. Demographic data and clinical measurements were collected, including age, sex, body mass index (BMI), smoking status and amount, respiratory symptoms, pulmonary function test (PFT), chest CT findings, sputum culture study, and comorbidities.

### 2.2. Sputum Sample Acquisition Method

Before the sputum acquisition, all patients were asked to rinse their mouth with sterile saline and to breathe deeply five times. Patients then, immediately, produced the sputum (≥1mL) by repeated deep breaths and coughing into a sterile container. In patients with no sputum, 5 cc of 3% NaCl was inhaled using a nebulizer and the induced sputum was collected for the study [9]. Acquired sputum samples were stored at −70 °C, in a freezer, and the DNA extraction was performed within 24 h, after the sputum acquisition. DNA extraction was performed with a commercial DNA extraction kit (PowerSoil DNA isolation kit, Mo Bio Laboratories, Inc. Carlsbad, CA, USA). Extracted DNA samples were stored at −20 °C, in a freezer, before the analysis by a polymerase chain reaction (PCR).

### 2.3. PCR Amplification and Sequencing

Purified DNA was used as a template for the PCR amplification with primers targeting the V3 and V4 regions of the 16S rRNA gene. The primers were 341F (5′-TCGTCGGCAGCGTC-AGATGTGTATAAGAGACAG-CCTACGGGNGGCWGCAG-3′) and 805R (5′-GTCTCGTGGGCTCGG-AGATGTGTATAAGAGACAG-GACTACHVGGGTATCTAATCC-3′). The amplification program was as follows. First, denaturation at 95 °C for 3 min was done, followed by 25 cycles of denaturation at 95 °C for 30 s. Primers were annealed at 55 °C for 30 s and extended at 72 °C for 30 s, using a final elongation at 72 °C for 5 min. To attach the Illumina NexTera barcode, a secondary amplification was carried out with the i5 forward primer (5′-AATGATACGGCGACCACCGAGATCTACAC-XXXXXXXX-TCGTCGGCAGCGTC-3′; X indicates the barcode region) and the i7 reverse primer (5′-CAAGCAGAAGACGGCATACGAGAT-XXXXXXXX-AGTCTCGTGGGCTCGG-3′). The program for the secondary amplification was the same as described above, except that the amplification cycle was set to eight.

Using 2% agarose gel electrophoresis and a Gel Doc system (BioRad, Hercules, CA, USA), the PCR amplification products were confirmed and then purified using the QIAquick PCR purification kit (Qiagen, Valencia, CA, USA). Short fragments (non-target products) were removed using the Ampure beads kit (Agencourt Bioscience, Waltham, MA, USA). The products were assessed on a Bioanalyzer 2100 (Agilent, Palo Alto, CA, USA) for quality and size, using a DNA 7500 chip.

Mixed amplicons were pooled and an Illumina MiSeq Sequencing system (Illumina, San Diego, CA, USA) was used for sequencing, which was performed at the Chun Lab, Inc. (Seoul, Korea), according to the manufacturer’s instructions [10].

### 2.4. Miseq Pipeline Method

To remove the low-quality reads, quality checks and the filtering of raw reads were performed by Trimmomatic 0.32 [11]. After the quality control, PANDAseq was used for merging the paired-end sequence data. With the help of the ChunLab’s program, primers were trimmed (cut off value: 0.8). Using the HMMER’s hmmsearch program, non-specific amplicons, which do not encode the 16S rRNA, were detected. The process of denoising the sequences was performed with the DUDE-Seq, and the non-redundant reads were extracted through UCLUST-clustering. Taxonomic assignments were obtained using USEARCH (8.1.1861_i86linux32), as implemented in the EzBioCloud database.

UCHIME^7^ and the non-chimeric 16S rRNA database from the EzBioCloud were used to find identify chimeras in the reads, with a best hit similarity rate of less than 97%. Sequence data were clustered using the CD-HIT^8^ and the UCLUST^5^. The alpha diversity indices and rarefaction curves were estimated using an in-house code.

### 2.5. Ethics Statement

The Institutional Review Board (IRB) of Seoul National University Bundang Hospital reviewed and approved this prospective study protocol (IRB approval number: B-1703/386-301). Informed written consent was obtained from the all patients on the day of sputum collection. All procedures were performed in accordance with the Declaration of Helsinki.

## 3. Results

The baseline characteristics of the study population are presented in Table 1. Age was higher (74.5 ± 5.9 years vs. 64.8 ± 9.0 years) and there were more cases of dyspnoea (33.3% vs. 7.1%), among patients with moderate/severe bronchiectasis, than among those with mild bronchiectasis (*p* < 0.001 and *p* = 0.012, respectively). Although the percentage of men and smokers was higher in the moderate/severe group, these differences were not significant (*p* = 0.285, and *p* = 0.114, respectively). Sputum was the most common respiratory symptom in the study population. Table 2 lists the comorbidities and the results of the pulmonary function tests. There were no significant differences in comorbidities between the two study groups. Non-tuberculosis mycobacterium (NTM) disease, which was included as a diagnosis, in 2007 by the American Thoracic Society (ATS)/Infectious Diseases Society of America (IDSA), was the most common comorbidity in both groups, but there was no significant difference in the NTM diseases between the two groups (*p* = 0.721). The prevalence of NTM was 52.4% in the mild bronchiectasis group, and 57.1% in the moderate/severe group. The moderate/severe group showed significantly reduced lung function. Forced vital capacity (FVC, %) was 75.3 ± 19.8 in the moderate/severe group and 88.4 ± 16.5 in the mild group (*p* = 0.007). FEV_1_ (%) was 66.7 ± 24.5 in the moderate/severe group and 88.0 ± 21.1 in the mild group (*p* = 0.001). The ratio of FEV1/FVC was also significantly lower in the moderate/severe group (*p* = 0.001). The value of the diffusing capacity for carbon monoxide (DLco) was within the normal range in both groups.

**Figure 2:**
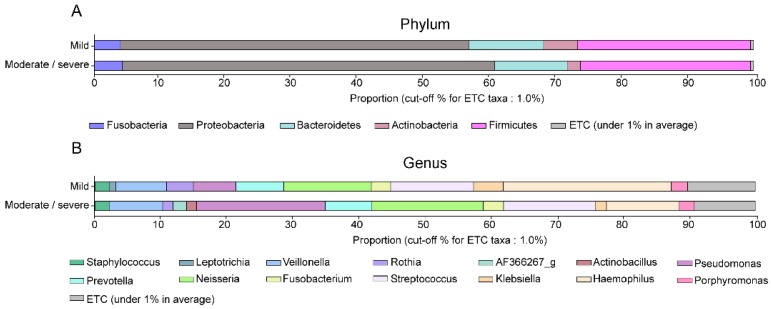
Abundance of the dominant bacteria in patients with bronchiectasis according to disease severity: (**A**) Phylum level, and (**B**) genus level. Haemophilus and Rothia were significantly more abundant in the mild bronchiectasis group than in the moderate/severe bronchiectasis group (*p* = 0.005, and *p* = 0.043, respectively), and Pseudomonas was significantly more common in the moderate/severe group (*p* = 0.031).

The dominant bacteria among the patients of the two study groups are shown in Table 3 and Figure 2. Proteobacteria and Firmicutes were the most common phyla. Although the percentage of Proteobacteria was higher in the moderate/severe bronchiectasis group and that of Actinobacteria was higher in the mild bronchiectasis group, there were no significant differences in relative abundance, at the phylum level, between the two study groups (Figure 2A). At the genus level, *Haemophilus* and *Rothia* were significantly more abundant in the mild bronchiectasis group than in the moderate/severe bronchiectasis group (*p* = 0.005, and *p* = 0.043, respectively), whereas *Pseudomonas* was significantly more common in the moderate/severe group (*p* = 0.031) (Figure 2B). *Mycobacterium* was detected in a few patients through the 16S rRNA gene sequencing analysis; *Mycobacterium*_uc_s was detected in three patients, while *Mycobacterium abscessus* and the *Mycobacterium bisbanense* complex were detected in one patient, each.

**Figure 3:**
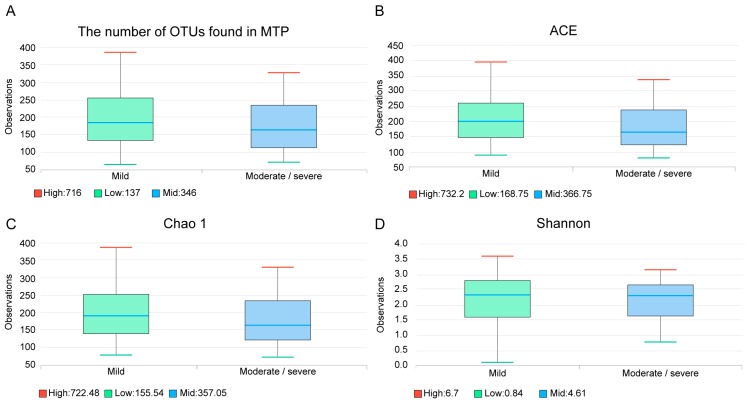
The number of operational taxonomic units ((**A**)*p* = 0.277) and species richness estimates in the two groups using ACE ((**B**), *p* = 0.274), Chao 1 ((**C**), *p* = 0.307), and Shannon diversity ((**D**), *p* = 0.550) indexes. OTU, operational taxonomic unit; ACE, abundance-based coverage estimator.

The median number of operational taxonomic unit (OTU) was 189 (Q1: 132, Q3: 252), in the mild bronchiectasis group, and 157 (112, Q1; 234, Q3) in the moderate/severe group; this difference was not significant (*p* = 0.277) (Figure 3A). Species richness estimates were not significantly different between the two groups, as demonstrated by the abundance-based coverage estimator (ACE, Figure 3B, *p* = 0.274) and Chao 1 index (Figure 3C, *p* = 0.307). The Shannon diversity index was also not significantly different (Figure 3D, *p* = 0.550).

**Figure 4:**
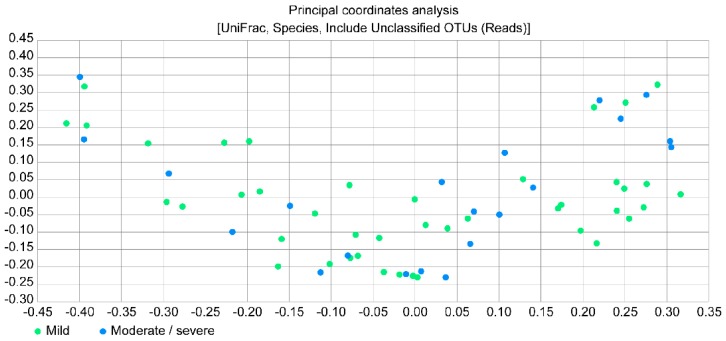
Principal coordinates analysis plot between the mild bronchiectasis group and the moderate/severe bronchiectasis group.

Figure 4 presents a principal coordinates analysis (PCoA) plot, which provides the beta diversity between the two study groups by estimating the relative distance; however, no significant difference was observed between the groups.

## 4. Discussion

In this study, we examined the sputum microbiota of bronchiectasis patients using NGS for the 16S rRNA gene pyrosequencing to determine the relationship between the microbiota composition and the bronchiectasis severity. Overall, culture-independent, DNA-based molecular techniques did not identify significant differences between patients with mild bronchiectasis and moderate or severe bronchiectasis. The OTU values and species richness estimates were not significantly different between the two groups. Only the abundance of the genera *Pseudomonas*, *Haemophilus*, and *Rothia* were significantly different between the two groups, according to DNA sequencing. Moreover, a significant difference was found in the detection of NTM, using either NGS-based analysis or culture growth-based methods. However, neither *Rothia* nor NTM affected the severity of bronchiectasis.

*P. aeruginosa* is the most common pathogen in patients with NTM disease [12]. In our study, the relative abundance of the genus *Pseudomonas* was significantly different between the mild and the moderate/severe bronchiectasis group. Therefore, we hypothesized that the proportion of NTM cases would be significantly higher in the moderate/severe bronchiectasis group than in the mild bronchiectasis group, but this was not confirmed by our data. This observation suggests that while bronchiectasis severity and progression are affected by the presence of *P. aeruginosa*, NTM itself may not have an effect on the bronchiectasis severity. Faverio et al. [13] compared bronchiectasis patients with pulmonary NTM and those with chronic *P. aeruginosa* infection, in a prospective study. Patients with bronchiectasis and pulmonary NTM tended to have cylindrical bronchiectasis and a low disease severity. Another study investigated the US Bronchiectasis Research Registry and showed that *Pseudomonas* was isolated more often from the NTM-uninfected patients with bronchiectasis [14]. These studies demonstrated that NTM is not directly related to the severity of the bronchiectasis. Interestingly, NTM strains were rarely found using the NGS-based analysis, in our study. This might have been due to the sensitivity of the method for detecting NTM; the NGS-based analysis might not yet be optimized for NTM detection, whereas in the acid-fast bacilli (AFB) tests, microbiologists are trained to identify NTM or tuberculosis, using the optimized growth conditions. This lack of optimization for NTM detection might be responsible for the difference in detection rates between the conventional culture method and the NGS-based analysis. Further large-scaled studies are needed to investigate the optimal method of NTM detection.

*Haemophilus* was the most common genus in our study, and its relative abundance was significantly higher in the mild bronchiectasis group, whereas that of *Pseudomonas* was significantly higher in the moderate/severe bronchiectasis group. King et al. [15] studied the longitudinal change in microbial organisms in right-nine patients with bronchiectasis, over 5.7 years. In their study, the relative abundance of the *H. influenza* was initially 47%, but this decreased to 40%, during the follow-up examination, whereas that of *P. aeruginosa* increased from 12% to 18%. In addition, the authors showed that the clinical severity of bronchiectasis was higher in patients with *P. aeruginosa* than in patients with *H. influenza*. The authors suggested that the disease progresses from no pathogen to *Haemophilus* to *Pseudomonas*.

*Rothia* was originally proposed and classified as a member of the Micrococcaceae family, by Georg & Bronwn in 1967 [16]. Lim et al. [17] found that *Rothia mucilaginosa* was prevalent in patients with cystic fibrosis that carried *P. aeruginosa*. Interestingly, there is no obvious pattern of synergy or competition between the two organisms. Previous studies have shown that *R. mucilaginosa* maybe a lower respiratory pathogen in both immunocompetent and immunocompromised patients [18,19,20]. *Rothia*, mostly *R. mucilaginosa*, was also a predominant organism in bronchiectasis, in our study. Although the proportion of *Rothia* was significantly higher in the mild bronchiectasis group, the abundance of *R. mucilanginosa* was not significantly different between the two groups (*p* = 0.064), similar to the findings of Lim et al.

Recently, Byun et al. [5] reported the characterization of the lung microbiome in stable or exacerbated bronchiectasis, using the bronchoalveolar fluid samples from fourteen patients. The authors found that *H. influenza*, *P. aeruginosa*, *M. catarrhalis*, and *Prevotella* spp. were common. Specifically, they suggested that *Prevotella* and *Veillonella* could be potent anaerobic pathogens. In our study, although *Prevotella* and *Veillonella* were common in both the mild and the moderate/severe bronchiectasis groups, the abundances of the two pathogens were not significantly different between the groups. This may indicate that *Prevotella* and *Veillonella* are risk factors for the exacerbation of bronchiectasis, but are not significantly associated with bronchiectasis severity. The authors also showed that the species richness, as estimated by the Simpson’s, and Shannon’s indices did not differ at the genus or the family level, between the clinically stable bronchiectasis group and the exacerbated bronchiectasis group. Similar to our study results, the number of OTUs, the ACE, Chao 1, and Shannon’s indices, and PCoA plot did not indicate significant differences between the mild bronchiectasis group and the moderate/severe bronchiectasis group.

There were some limitations to our study. First, although we used a previously validated method to acquire the high-quality samples, any sample could have become contaminated while passing through the oral space. Second, although the DNA sequencing 16S rRNA analysis is sensitive and more informative than the conventional, culture-based methods, it is limited with regards to the amplification primer. Only well-known binding sites can be used for the pyrosequencing platforms. Third, daily diet and antibiotic use of patients was not investigated in this study. If this information would be available, results of this study would be more informative, with respect to patient history and the dynamics of the lung microbiome [21].

## 5. Conclusions

In conclusion, although the abundance of *Haemophilus* and *Rothia* differed, significantly, in relation to the severity of bronchiectasis, the NGS-based technique did not identify significant differences between the alpha diversity and the beta diversity of the lung microbiomes of the mild bronchiectasis group and the moderate/severe bronchiectasis group. Respiratory microbial community in bronchiectasis consisted of several abundant genera that did not significantly differ in relation to disease severity. Further prospective large-scale studies are needed to investigate the microbiome in bronchiectasis.

## Figures and Tables

**Table 1:** Baseline characteristics of the study population according to the severity of bronchiectasis.

	Mild (*n* = 42)	Moderate or Severe (*n* = 21)	*p*-Value
Age (years)	64.8 ± 9.0	74.5 ± 5.9	<0.001
Sex, male (%)	18 (42.9)	12 (57.1)	0.285
BMI (kg/m^2^)	22.0 ± 3.2	22.3 ± 3.5	0.783
Smoking status			0.604
Never smoker	29 (69.0)	13 (61.9)	
Ex-smoker	12 (28.6)	8 (38.1)	
Current smoker	1 (2.4)	-	
Smoking amount (pack-year)	4.3 ± 11.1	10.7 ± 19.4	0.114
Respiratory symptom			
Dyspnea	3 (7.1)	7 (33.3)	0.012
Cough	14 (33.3)	9 (42.9)	0.580
Sputum	25 (59.5)	8 (38.1)	0.108
Hemoptysis	1 (2.4)	2 (10.0)	0.241

Abbreviations: BMI; body mass index.

**Table 2:** Comorbidities and clinical data according to the severity of bronchiectasis.

	Mild (*n* = 42)	Moderate or Severe (*n* = 21)	*p*-Value
Comorbidities			
Diabetes mellitus	1 (2.4)	3 (14.3)	0.104
Hypertension	10 (23.8)	6 (28.6)	0.682
Gastroesophageal reflux disease	8 (19.0)	4 (19.0)	1.000
Sinusitis	5 (11.9)	2 (9.5)	1.000
Cardiovascular disease	1 (2.4)	1 (4.8)	1.000
Stroke	3 (7.1)	0 (0.0)	0.545
Liver disease	2 (4.8)	2 (9.5)	0.595
Renal disease	1 (2.4)	1 (4.8)	1.000
Non-tuberculosis mycobacterium	22 (52.4)	12 (57.1)	0.721
Pulmonary function			
FVC (%)	88.4 ± 16.5	75.3 ± 19.8	0.007
FEV_1_ (%)	88.0 ± 21.1	66.7 ± 24.5	0.001
FEV_1_/FVC ratio	0.71 ± 0.09	0.60 ± 0.16	0.001
DL_CO_ (%)	103.3 ± 22.9	92.8 ± 21.7	0.170

**Table 3:** Abundance of specific bacteria in bronchiectasis, by severity.

Classification	Mild (*n* = 42)			Moderate or Severe (*n* = 21)			*p*-Value
	Total Reads	%	Occurred	Total Reads	%	Occurred	
Phylum							
Proteobacteria	32,780	50.9	42	31,405	57.0	21	0.814
Firmicutes	16,119	25.1	42	13,090	23.8	21	0.431
Bacteroidetes	8300	12.9	42	6555	11.9	21	0.499
Actinobacteria	3893	6.1	42	1311	2.4	21	0.099
Fusobacteria	2726	4.2	42	2346	4.3	21	0.722
Saccharibacteria_TM7	293	0.5	36	137	0.2	18	0.259
Spirochaetes	146	0.2	35	128	0.2	17	0.862
Tenericutes	48	0.1	24	41	0.1	12	0.842
>Synergistetes	>10	>0.0	>24	>43	>0.1	>15	>0.227
Genus							
Haemophilus	15,663	24.3	42	5767	10.5	21	0.005
Neisseria	8828	13.7	42	10,405	18.9	21	0.618
Streptococcus	7439	11.6	42	6830	12.4	21	0.817
Pseudomonas	3082	4.8	39	9408	17.1	18	0.031
Veillonella	5496	8.5	42	4150	7.5	21	0.444
Prevotella	5243	8.1	42	3830	7.0	21	0.395
Rothia	3181	4.9	42	1089	2.0	21	0.043
Klebsiella	3279	5.1	28	933	1.7	11	0.386
Fusobacterium	1865	2.9	42	1928	3.5	21	0.950
Porphyromonas	1744	2.7	41	1640	3.0	20	0.926
Actinobacillus	544	0.8	34	1265	2.3	19	0.441
Staphylococcus	766	1.2	26	1077	2.0	15	0.810
Leptotrichia	798	1.2	39	404	0.7	19	0.079
AF366267_g	12	0.0	27	1736	3.2	13	0.325
